# Triglyceride-glucose index as a biochemical predictor of hypertension in patients with type 2 diabetes and prediabetes: Evidence from the NHANES database

**DOI:** 10.5937/jomb0-61307

**Published:** 2026-02-27

**Authors:** Tianyu Dai, Fangze Wang, Hanqing Deng, Muyuan Li, Jihu Zhang, Jian Wang

**Affiliations:** 1 Shandong Second Medical University, School of Clinical Medicine, Weifang, China; 2 Shandong Second Medical University, Department of Cardiology, Weifang People's Hospital, Weifang, China

**Keywords:** triglyceride-glucose index, biochemical marker, hypertension, type 2 diabetes, NHANES, triglicerid-glukozni indeks, biohemijski marker, hipertenzija, dijabetes tipa 2, NHANES

## Abstract

**Background:**

The triglyceride-glucose (TyG) index, derived from fasting triglyceride and glucose levels, has emerged as a reliable biochemical marker of insulin resistance. However, its relationship with hypertension (HTN) in diabetic and prediabetic populations remains insufficiently studied.

**Methods:**

Using data from 2,440 adults in the U.S. National Health and Nutrition Examination Survey (NHANES, 2011-2018), we investigated the association between the TyG index and HTN. Biochemical assays included enzymatic measurement of serum triglycerides (Beckman Coulter DxC800) and plasma glucose (hexokinase-based methods on Roche Cobas systems), from which the TyG index and related indices (TyG-BMI, TyG-WC, TyG-WHtR) were calculated. Logistic regression models adjusted for demographic, anthropometric, and biochemical covariates were used to estimate odds ratios (OR) for HTN risk.

**Results:**

Participants with HTN exhibited significantly higher TyG index levels compared with non-hypertensives (8.44 vs. 8.38, P&lt; 0.01). In fully adjusted models, each unit increase in TyG was associated with a 31% higher risk of HTN (OR = 1.31, 95% CI: 1.02-1.69). Stratified analyses revealed significant associations among Mexican Americans and individuals without prior cardiovascular or hepatic disease. TyG-WC and TyG-WHtR indices also correlated positively with HTN risk, whereas TyG-BMI did not.

**Conclusions:**

Elevated TyG index and related biochemical indices are independently associated with increased HTN risk among individuals with diabetes or prediabetes. As a simple and reproducible biochemical parameter, the TyG index may serve as a valuable tool in laboratory medicine for early HTN risk identification and prevention strategies.

## Abstract (sr)

**Uvod:**

Triglicerid-glukozni (TyG) indeks, izveden iz nivoa triglicerida i glukoze na prazan stomak, pojavio se kao pouzdan biohemijski marker insulinske rezistencije. Međutim,njegova veza sa hipertenzijom (HTN) kod dijabetičarskih ipredijabetičnih populacija jos uvek nije dovoljno proučena.

**Metode:**

Koristeći podatke od 2.440 odraslih u okviruNacionalnog istraživanja o zdravlju i ishrani SAD (NHANES, 2011-2018), istražili smo vezu između TyG indeksa ihipertenzivne neuralgije (HNN). Biohemijska ispitivanja suobuhvatala enzimsko merenje serumskih triglicerida(Beckman Coulter DxC800) i glukoze u plazmi (metodezasnovane na heksokinazi na Roche Cobas sistemima), izkojih su izračunati TyG indeks i povezani indeksi (TyG-BMI,TyG-WC, TyG-WHtR). Modeli logističke regresije prilagođeni za demografske, antropometrijske i biohemijske kovarijate korisceni su za procenu odnosa sansi (OR) za rizik odHNN.

**Rezultati:**

Učesnici sa hipertenzijom (HTN) pokazali su značajno vise nivoe TyG indeksa u poređenju sa osobama bezhipertenzije (8,44 naspram 8,38, P&lt;0,01). U potpuno prilagođenim modelima, svako povećanje TyG-a za jedinicu biloje povezano sa 31% većim rizikom od HTN (O R=1,31, 95%CI: 1,02-1,69). Stratifikovane analize su otkrile značajnepovezanosti između meksičkih Amerikanaca i osoba bezprethodnih kardiovaskularnih ili hepatičnih bolesti. IndeksiTyG-WC i TyG-WHtR takođe su pozitivno korelirali sa rizikomod HTN, dok TyG-BMI nisu.

**Zaključak:**

Povišen TyG indeks i povezani biohemijski indeksisu nezavisno povezani sa povecanim rizikom od hipertenzije(HTN) kod osoba sa dijabetesom ili predijabetesom. Kaojednostavan i reproducibilan biohemijski parametar, TyGindeks može poslužiti kao vredan alat u laboratorijskoj medicini za rano otkrivanje rizika od HTN i strategije prevencije.

## Introduction

HTN, a widespread chronic condition among adults, is acknowledged as the foremost modifiable risk factor for cardiovascular diseases (CVD) [1]. Every year, it claims the lives of almost nine million individuals and affects nearly 100 million Americans [2]. HTN, sometimes called the »silent killer,« greatly increases the risk of CVD, stroke, kidney disease, and other serious health problems despite the fact that it usually shows no outward symptoms. Although there have been notable advancements in the diagnosis and treatment of HTN, its complex origins and varied presentations continue to challenge effective management and control. Research has demonstrated that dyslipidemia significantly increases the likelihood of HTN development [3][4]. Dyslipidemia, characterized by unusually high levels of triglycerides (TG), elevated total cholesterol (TC), increased low-density lipoprotein cholesterol (LDL-C), and reduced high-density lipoprotein cholesterol (HDL-C) [5][6][7][8], is associated with HTN. Atherosclerosis, a disorder that greatly affects artery functionality and structure, can make it harder to regulate blood pressure (BP) and increase the risk of developing HTN [9][10][11].

Hyperglycemia, caused by the body's inability to regulate blood glucose, is a hallmark of Diabetes Mellitus (DM). When the pancreas' β-cells are autoimmunely destroyed, the inability to produce enough insulin leads to Type 1 Diabetes (T1D). Type 2 Diabetes (T2D), on the other hand, has its roots in IR [12]. All age groups are affected by diabetes, which is a reversible risk factor for many other diseases and conditions. An increase in the prevalence and interaction between HTN and T2D is facilitated by IR [13]. High BP is seen in a large percentage of people with T2D [14]. HTN also complicates the management of diabetes and aggravates associated microvascular and macrovascular complications, such as nephropathy, retinopathy, renal failure, and coronary artery disease [15]. Thus, the early identification of HTN potential in those with diabetes and prediabetes is crucial [16]. The TyG index, integrating measurements of fasting triglycerides and glucose [17][18], offers a straightforward and effective method for IR assessment and is a reliable predictor of cardiovascular diseases [19]. Nonetheless, existing studies on the TyG index's correlation with HTN in diabetic and prediabetic populations are sparse, necessitating further investigation with larger cohorts.

The association between the TyG index and HTN in individuals with diabetes or prediabetes was investigated by analyzing data from 2440 American participants of the NHANES from 2011 to 2018. Our goal was to fill this research gap. Theoretically, this study intends to help identify individuals with diabetes and prediabetes who may have a higher likelihood of having HTN.

## Materials and methods

### Study population

The NHANES database facilitates an assessment of the nutritional and health statuses of the U.S. population, drawing on a meticulously structured multistage stratified sampling approach. This cross-sectional study synthesizes data from various sources including questionnaires, physical examinations, assessments of dietary intake, and laboratory tests, coordinated by the National Center for Health Statistics (NCHS) of the CDC. Ethical approval for the study was granted by the NCHS Institutional Review Board, with all participants providing signed informed consent following comprehensive briefings [20][21]. The study was approved by the NCHS Institutional Review Board, and after a thorough briefing, all participants gave their signed informed permission. Numerous research projects rely on NHANES data, which is accessible to the public online.

**Figure 1:**
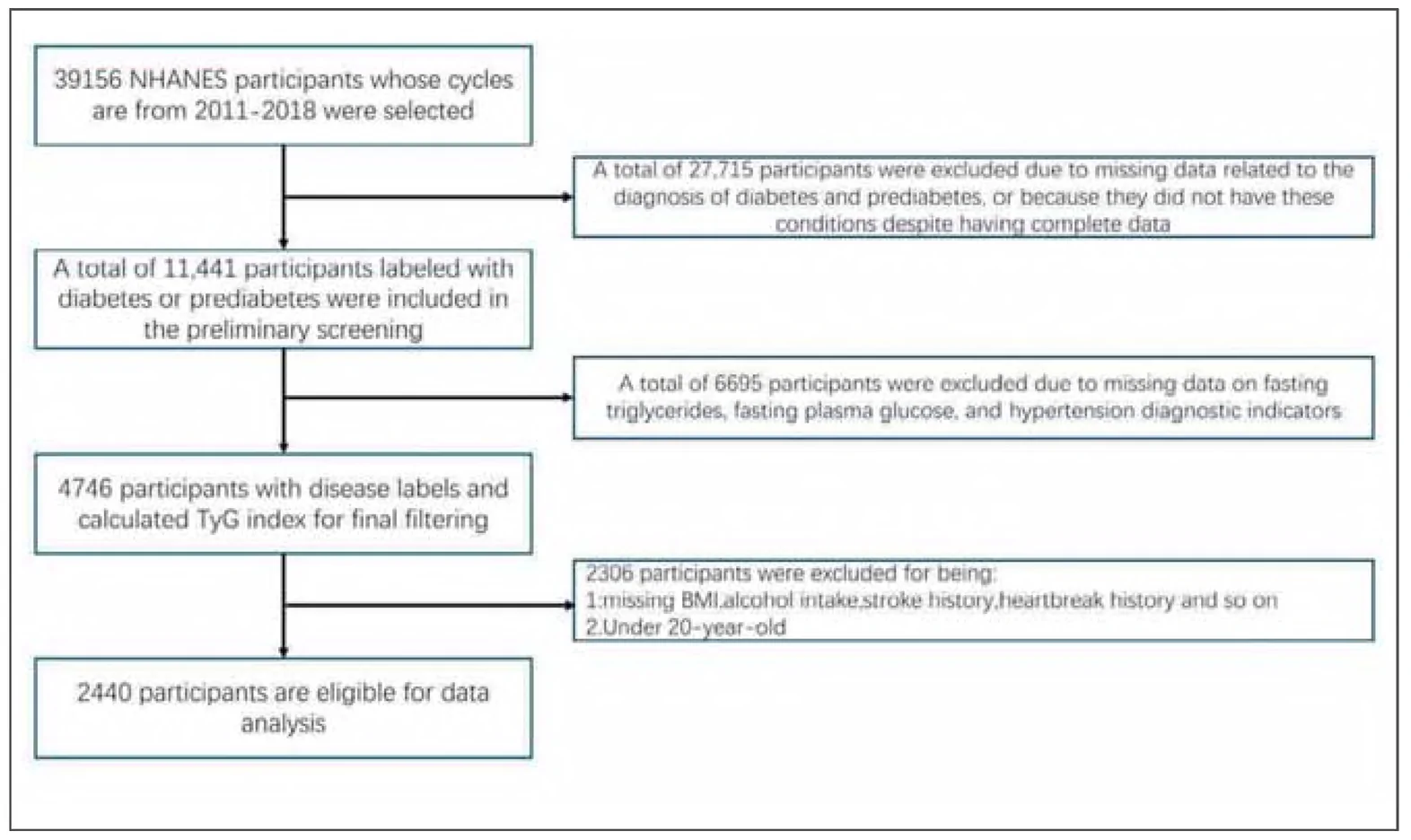
Flowchart of participant selection.

Our study population was drawn from four cycles of the National Health and Nutrition Examination Survey (NHANES) between 2011 and 2018, which initially included 39,156 participants. Our selection process followed a clear, sequential pathway. First, we applied our primary inclusion criterion, identifying all participants who met the diagnostic criteria for diabetes or prediabetes as defined in the 'Covariates' section. This step excluded 27,715 individuals who did not have either condition, resulting in a target cohort of 11,441 individuals. Subsequently, we applied a series of exclusion criteria to this cohort. We first removed 1,290 participants who had incomplete data for hypertension (HTN) diagnosis, followed by an additional 5,405 participants who lacked the necessary fasting plasma glucose or triglyceride values to calculate the Triglyceride-Glucose (TyG) index. Finally, a further 2,306 participants were excluded because they were younger than 20 years of age or had missing data for any of the covariates included in our fully adjusted model, specifically: age, sex, race, education, PIR, alcohol consumption, smoking status, height, weight, waist circumference, BMI, serum albumin, creatinine, uric acid, iron, potassium, sodium, calcium, total cholesterol, LDL-C, and history of CHD, stroke, myocardial infarction, or liver disease. After applying these criteria, a final sample of 2,440 participants was included in our analysis, as detailed in the flowchart (Figure 1).

### Outcome Variable: Hypertension (HTN)

Blood pressure (BP) was measured by trained technicians with participants seated, using a mercury-calibrated sphygmomanometer on the right upper arm. Up to three consecutive readings were taken, and the average was calculated for analysis. HTN was defined as meeting any of the following three criteria: (1) an average systolic BP ≥130 mmHg or an average diastolic BP ≥80 mmHg, based on the 2017 ACC/AHA guidelines [22]; (2) self-reported use of antihypertensive medication; or (3) a self-reported physician diagnosis of hypertension. This comprehensive definition ensures that individuals with a history of hypertension whose BP is controlled by medication are correctly classified.

### Exposure variable: triglyceride-glucose (TyG) index and related parameters

The triglyceride-glucose index is derived by computing the natural logarithm of the product of fasting triglyceride and fasting glucose levels, utilizing the formula ln((triglyceride in mg/dL) X (fasting glucose in mg/dL)/2 [23][24]. In NHANES, biochemical parameters were measured under standardized protocols. Serum triglycerides were quantified by a timed endpoint method using the DxC800 clinical chemistry analyzer (Beckman Coulter). Fasting plasma glucose was determined using a hexokinase-based enzymatic assay performed on either the Roche Cobas C311 or Roche/Hitachi Cobas C501 chemistry analyzers. These methods ensured high analytical precision and reproducibility, in accordance with CDC quality-control procedures [25].

Derived TyG-related indices were computed as follows [25]: TyG-BMI = TyG index X body mass index (BMI); TyG-WC = TyG index X waist circumference (WC); TyG-WHtR = TyG index X waist-to-height ratio (WHtR).

### Covariates

Demographic, anthropometric, medical, and biochemical factors were considered as covariates. Demographic variables included age, sex, race/ethnicity, educational attainment, and family income-to-poverty ratio (PIR). Anthropometric data (height, weight, WC) were measured by trained professionals in the Mobile Examination Center (MEC). BMI was calculated as weight (kg)/height (m^2^) and classified into 25, 25-30, and >30 kg/m^2^.

Medical history included self-reported diagnoses of stroke, liver disease, coronary heart disease (CHD), or myocardial infarction [26][27][28]. Type 2 diabetes (T2D) was defined as: (1) fasting plasma glucose ≥126 mg/dL; (2) insulin use; (3) HbA1c ≥6.5%; or (4) physician's diagnosis of diabetes [29]. Prediabetes was defined as HbA1c 5.7-6.4% or fasting glucose 100-125 mg/dL, or self-reported prediabetes [30].

Health behaviors included smoking (≥100 lifetime cigarettes) and alcohol consumption (categorized by standard thresholds) [19].

Biochemical covariates were measured in the N HAN ES laboratory under rigorous CDC protocols and included: serum albumin, electrolytes (Na^+^, K^+^, Ca^2+^), iron, uric acid, lipid profile [total cholesterol (TC), low-density lipoprotein cholesterol (LDL-C)], serum creatinine (SCR), and blood urea nitrogen (BUN). These biochemical markers were included to capture renal, metabolic, and cardiovascular-related confounding factors.

### Statistical analysis

There was strict adherence to Centers for Disease Control (CDC)-specified methods in all statistical analyses. The complex multistage cluster architecture of the survey was taken into account in the analyses, which also included suitable NHANES sample weights. Categorical data was represented by percentages, whereas continuous variables were shown by means and standard deviations (SD). To compare groups that were divided according to TyG quartiles, researchers utilized weighted Student's t-tests or weighted Chi-Square testing. The chi-square test was employed for categorical variables, while the t-test was used for continuous variables. To investigate the association between the TyG index and the odds of having HTN in people who have diabetes or prediabetes, we employed multivariate logistic regression analysis. Unlike HTN, which was considered a binary variable, the TyG index was considered a continuous one. The logistic regression analysis utilized three distinct models. No adjustments were made to the covariates in Model I. Variables such as sex, age, race, alcohol intake, and smoking status were modified in Model II. Model III underwent thorough revisions that encompassed a wide range of demographic variables, such as sex, age, race, education, poverty income ratio (PIR), alcohol intake, smoking history, height, weight, waist circumference, body mass index, and a battery of serum measurements, including albumin, creatinine, uric acid, iron, potassium, sodium, and calcium. Model III also took into account total cholesterol, low-density lipoprotein cholesterol, and a patient's history of cardiovascular illness, stroke, heart attack, and liver disorders. By dividing the subjects into subgroups based on age, gender, race, alcohol consumption, smoking status, BMI, and history of disease, stroke, hepatitis, and other factors, we were able to examine the link between the TyG index and HTN. These parameters were also considered as potential moderators of effect that had already been described. To assess the dispersion of relationships among categories, an interaction term was incorporated. When dealing with missing data, the existing dataset mandated that continuous variables be treated as median and categorical variables as mode. Statistical analyses were conducted using R 3.4.3 and Empower 3.2.2. Significance was set at p<0.05.

## Results

### Baseline characteristics

Within 2, 440 participants, the average age was 53.19 years, with females comprising 43.52% and 81.15% aged below 69 years. The demographic breakdown revealed that 37.83% were non-Hispanic whites. Lifestyle analysis indicated that 24.43% of participants were heavy drinkers and 46.93% had smoked over 100 cigarettes in their lifetime. Educational attainment was high, with 56.8% having achieved a college education or higher. A significant majority, 81.27%, fell into the overweight or obese categories. Regarding health history, the vast majority reported no previous occurrences of CHD (94.75%), stroke (95.49%), myocardial infarction (MI) (94.22%), or liver disease (94.47%). The cohort's mean TyG index was 8.41, stratified into quartiles as follows: Quartile 1 ranged from 7.47 to 8.05, Quartile 2 from 8.16 to 8.36, Quartile 3 from 8.49 to 8.67, and Quartile 4 from 8.79 to 9.19. Significant statistical differences (p<0.05) across these quartiles were noted in variables such as age, BMI, weight, waist circumference, levels of blood LDL, TC, sodium, BUN, uric acid, along with the prevalence of CHD, MI, stroke, and liver disease. Individuals in the higher TyG quartiles faced greater risks concerning weight, BMI, BUG levels, and aforementioned medical conditions. Notably, their levels of blood LDL and TC were also reduced. No significant differences were found in gender, race, alcohol intake, smoking habits, or the ratio of PIR across quartiles. Detailed baseline characteristics of all participants from the 2011 to 2018 NHANES database are presented in Table 1.

**Table 1:** Baseline Characteristics of Participants from 2011 to 2018. Notes: Categorical variables are presented as n (%), and continuous variables are presented as mean (sd)

TyG index quartile	Q1	Q2	Q3	Q 4	P-value
N	603	599	626	612	
TyG index	7.76±0.29	8.26±0.10	8.58±0.09	8.99±0.20	<0.001
PIR	2.73±1.61	2.59±1.58	2.61±1.58	2.56±1.56	0.230
Weight (kg)	87.70±23.09	86.01±22.23	86.91±22.50	89.68±22.86	0.034
Height (cm)	168.59±9.92	168.19±10.08	168.14±9.71	167.75±9.54	0.526
Waist (cm)	103.78±16.50	103.37±16.19	104.02±16.22	107.44±16.22	<0.001
Albumin (mg/dL)	4.21±0.35	4.20±0.33	4.19±0.34	4.18±0.35	0.447
BUN (mg/dL)	14.08±4.86	14.32±5.69	14.59±5.77	15.26±6.66	0.003
LDL (mg/dL)	115.84±35.85	118.29±35.67	115.75±34.57	105.08±37.20	<0.001
TC (mg/dL)	193.87±42.32	196.00±40.39	192.56±40.86	181.86±42.90	<0.001
Ca (mmol/L)	2.34±0.09	2.34±0.08	2.34±0.09	2.34±0.10	0.869
Iron (umol/L)	15.06±5.90	15.49±5.88	15.11±6.36	14.75±5.91	0.201
Na (mmol/L)	139.46±2.31	139.47±2.59	139.41±2.40	138.70±2.85	<0.001
K (mmol/L)	4.01±0.35	4.05±0.36	4.01±0.37	4.02±0.39	0.352
Uric acid (mg/dL)	5.67±1.44	5.49±1.42	5.77±1.47	5.71±1.50	0.005
Gender					0.890
Male	343 (56.88%)	332 (55.43%)	360 (57.51%)	343 (56.05%)	
Female	260 (43.12%)	267 (44.57%)	266 (42.49%)	269 (43.95%)	
Age categorical (year)					0.002
<45	210 (34.83%)	197 (32.89%)	191 (30.51%)	158 (25.82%)	
>=45, <69	308 (51.08%)	286 (47.75%)	305 (48.72%)	325 (53.10%)	
>=6 9	85 (14.10%)	116 (19.37%)	130 (20.77%)	129 (21.08%)	
Race					0.801
Mexican American	83 (13.76%)	88 (14.69%)	105 (16.77%)	91 (14.87%)	
Other Hispanic	67 (11.11%)	61 (10.18%)	60 (9.58%)	77 (12.58%)	
Non-Hispanic White	224 (37.15%)	231 (38.56%)	234 (37.38%)	234 (38.24%)	
Non-Hispanic Black	145 (24.05%)	140 (23.37%)	143 (22.84%)	123 (20.10%)	
Other Race - Including Multi­-Racial	84 (13.93%)	79 (13.19%)	84 (13.42%)	87 (14.22%)	
Education					0.575
Less than high school	113 (18.74%)	122 (20.37%)	134 (21.41%)	135 (22.06%)	
High school or equivalent	137 (22.72%)	129 (21.54%)	153 (24.44%)	131 (21.41%)	
College or above	353 (58.54%)	348 (58.10%)	339 (54.15%)	346 (56.54%)	
Alcohol consumption					0.926
Mild drinker	329 (54.56%)	325 (54.26%)	345 (55.11%)	344 (56.21%)	
Moderate drinker	126 (20.90%)	121 (20.20%)	136 (21.73%)	118 (19.28%)	
Heavy drinker	148 (24.54%)	153 (25.54%)	145 (23.16%)	150 (24.51%)	
BMI categorical (kg/m^2^)					0.009
<25	113 (18.74%)	133 (22.20%)	120 (19.17%)	91 (14.87%)	
>=25, <30	219 (36.32%)	196 (32.72%)	225 (35.94%)	198 (32.35%)	
>=30	271 (44.94%)	270 (45.08%)	281 (44.89%)	323 (52.78%)	
Coronary heart disease					0.005
Yes	27 (4.48%)	21 (3.51%)	32 (5.11%)	48 (7.84%)	
No	576 (95.52%)	578 (96.49%)	594 (94.89%)	564 (92.16%)	
Heart attack					0.017
Yes	28 (4.64%)	26 (4.34%)	37 (5.91%)	50 (8.17%)	
No	575 (95.36%)	573 (95.66%)	589 (94.09%)	562 (91.83%)	
Stroke					0.008
Yes	21 (3.48%)	19 (3.17%)	28 (4.47%)	42 (6.86%)	
No	582 (96.52%)	580 (96.83%)	598 (95.53%)	570 (93.14%)	
Liver condition					<0.001
Yes	21 (3.48%)	28 (4.67%)	31 (4.95%)	55 (8.99%)	
No	582 (96.52%)	571 (95.33%)	595 (95.05%)	557 (91.01%)	
Smoking more than 100<br>cigarettes in one's lifetime					0.330
Yes	281 (46.60%)	265 (44.24%)	296 (47.28%)	303 (49.51%)	
No	322 (53.40%)	334 (55.76%)	330 (52.72%)	309 (50.49%)	

An analysis focusing on the prevalence of HTN served as the basis for categorizing characteristics (Table 2). The prevalence of HTN was found to be 45.29%, with significant differences (P<0.05) observed between groups in TyG index, gender, age, race, education level, weight, waist circumference, and specific blood biochemistry parameters such as potassium and uric acid. Among the hypertensive group, which included individuals with diabetes or prediabetes, 59.46% were male, and 54.48% were aged between 45-69 years. Notably, hypertensive subjects exhibited a higher TyG index than their non-hypertensive counterparts (8.44 versus 8.38, P<0.05).

**Table 2:** Characteristics of the Population by HTN Groups. Notes: Categorical variables are presented as n (%), and continuous variables are presented as mean (sd)

Hypertension	NO	YES	P-value
N	1335	1105	
TyG index	8.38±0.49	8.44±0.48	0.003
PIR	2.65±1.61	2.59±1.55	0.359
Weight (kg)	86.08±20.70	89.38±24.80	<0.001
Height (cm)	168.15±9.87	168.19±9.74	0.908
Waist (cm)	102.96±15.78	106.71±16.80	<0.001
Albumin (mg/dL)	4.21±0.34	4.19±0.34	0.099
BUN (mg/dL)	14.42±5.72	14.75±5.88	0.160
LDL (mg/dL)	113.95±36.31	113.43±36.01	0.722
TC (mg/dL)	190.93±41.89	191.18±42.06	0.883
Ca (mmol/L)	2.34±0.09	2.35±0.09	0.143
Iron (μmol/L)	14.94±6.06	15.30±5.97	0.142
Na (mmol/L)	139.20±2.49	139.33±2.65	0.226
K (mmol/L)	4.01±0.35	4.04±0.40	0.018
Uric acid (mg/dL)	5.53±1.45	5.82±1.45	<0.001
Gender			0.007
Male	721 (54.01%)	657 (59.46%)	
Female	614 (45.99%)	448 (40.54%)	
Age categorical (year)			<0.001
<45	524 (39.25%)	232 (21.00%)	
>=45, <69	622 (46.59%)	602 (54.48%)	
>=69	189 (14.16%)	271 (24.52%)	
Race			<0.001
Mexican American	232 (17.38%)	135 (12.22%)	
Other Hispanic	138 (10.34%)	127 (11.49%)	
Non-Hispanic White	505 (37.83%)	418 (37.83%)	
Non-Hispanic Black	267 (20.00%)	284 (25.70%)	
Other Race - Including Multi-Racial	193 (14.46%)	141 (12.76%)	
Education			0.019
Less than high school	252 (18.88%)	252 (22.81%)	
High school or equivalent	293 (21.95%)	257 (23.26%)	
College or above	790 (59.18%)	596 (53.94%)	
Alcohol consumption			0.116
Mild drinker	731 (54.76%)	612 (55.38%)	
Moderate drinker	259 (19.40%)	242 (21.90%)	
Heavy drinker	345 (25.84%)	251 (22.71%)	
BMI categorical (kg/m^2^)			0.127
<25	262 (19.63%)	195 (17.65%)	
>=25, <30	471 (35.28%)	367 (33.21%)	
>=30	602 (45.09%)	543 (49.14%)	
Coronary heart disease			0.580
Yes	67 (5.02%)	61 (5.52%)	
No	1268 (94.98%)	1044 (94.48%)	
Heart attack			0.111
Yes	68 (5.09%)	73 (6.61%)	
No	1267 (94.91%)	1032 (93.39%)	
Stroke			0.816
Yes	59 (4.42%)	51 (4.62%)	
No	1276 (95.58%)	1054 (95.38%)	
Liver condition			0.104
Yes	83 (6.22%)	52 (4.71%)	
No	1252 (93.78%)	1053 (95.29%)	
Smoking more than 100 cigarettes in one's lifetime			0.094
Yes	647 (48.46%)	498 (45.07%)	
No	688 (51.54%)	607 (54.93%)	

### The association between TyG index and HTN

Table 3 Explains how the TyG index is related to HTN. A greater TyG index was associated with higher odds of HTN, suggesting a strong connection.

**Table 3:** With full variable adjustment, ORs and 95% CI of the TyG-index quartiles associated with HTN. Notes: In sensitivity analysis, the TyG index was stratified into quartiles. 95% CI, 95% Confidence Interval; OR, Odds Ratio

Outcome: HTN	(N) % (95%CI)	OR (95%CI) P-value
TyG-index	(2440) 42.99 (40.41, 45.58)	1.31 (1.02, 1.69) 0.0440
TyG-index quartile		
Q1	(603) 40.89 (35.24, 46.54)	Ref.
Q2	(599) 37.14 (32.31, 41.97)	0.88 (0.63, 1.23) 0.4753
Q3	(626) 45.81 (40.71, 50.90)	1.26 (0.92, 1.70) 0.1566
Q4	(612) 48.52 (42.70, 54.34)	1.45 (1.01, 2.08) 0.0467
P for trend	(2440) 42.99 (40.41, 45.58)	1.15 (1.02, 1.30) 0.0253

According to the fully adjusted model, the risk of HTN increased by 31% for every unit increase in the TyG index (Model III: OR=1.31, 95% CI 1.02-1.69). This association was still statistically significant following quartile stratification of the TyG score. People in the top fourth of the TyG score had a 45% higher chance of developing HTN compared to those in the bottom fourth (OR=1.45, 95% CI 1.01-2.08, P<0.05).

### Subgroup analysis

**Figure 2:**
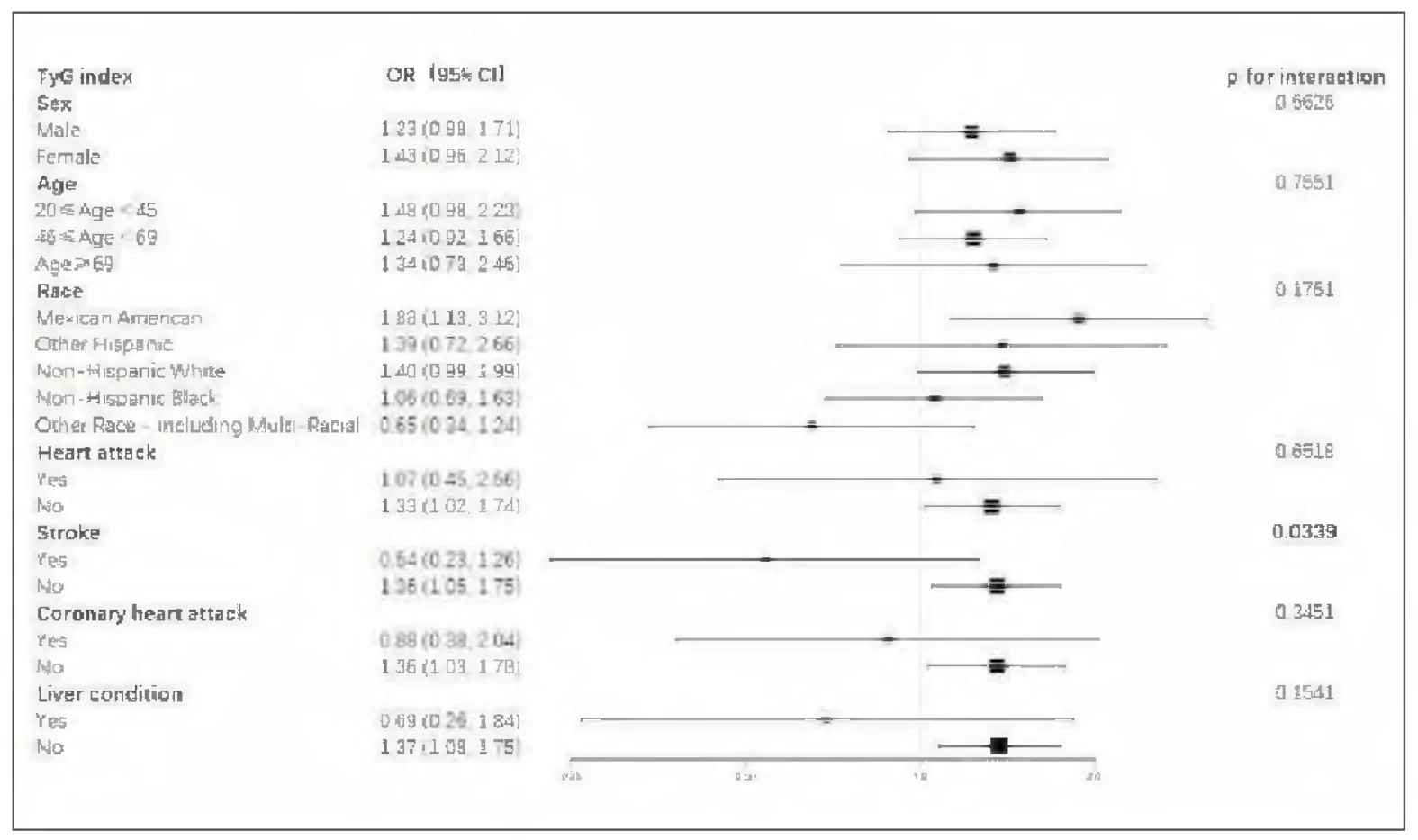
Subgroup analyses of the TyG-index and HTN in patients with type 2 diabetes or prediabetes.

Subgroup analyses were conducted on patients with diabetes or prediabetes to explore potential modifiers of the relationship between the TyG index and HTN risk. The influence of prior stroke was notably underscored in Figure 2, which also highlighted significant interactions (P < 0.05). Among Mexican Americans with these conditions, higher TyG index values were significantly linked to an increased HTN risk (OR > 1, P < 0.05), even when adjusting for all covariates. A unit increment in the TyG index was associated with an 88% heightened risk of HTN. Furthermore, the TyG index consistently exhibited a positive association with HTN risk among individuals without a history of MI, stroke, CHD, or liver disease (OR > 1, P < 0.05). The analyses indicated that this correlation was stable across different ages and genders.

Consequently, further investigations were conducted to assess how TyG-related indices - TyG-BMI, TyG-WC, and TyG-WHtR—correlate with HTN in the diabetic and prediabetic cohorts (Table 4). In the fully adjusted third model, TyG-WC and TyG-WHtR were positively associated with an increased risk of HTN (OR > 1, P < 0.05), while TyG-BMI did not demonstrate such a relationship. These relationships persisted in both partially adjusted and unadjusted models, evidencing a positive correlation with HTN risk (OR > 1, P < 0.05).

**Table 4:** ORs and 95% CI for TyG Index Parameters in Relation to Hypertension Across Different Models.

TyG index related parameters	Model	OR (95%CI)	P-value
TyG-BMI	Model I	1.00 (1.00, 1.01)	0.0002
	Model II	1.00 (1.00, 1.01)	<0.0001
	Model III	1.00 (1.00, 1.01)	0.2308
TyG-WC	Model I	1.00 (1.00, 1.00)	<0.0001
	Model II	1.00 (1.00, 1.00)	<0.0001
	Model III	1.00 (1.00, 1.00)	0.0384
TyG-WHtR	Model I	1.35 (1.19, 1.54)	<0.0001
	Model II	1.42 (1.24, 1.63)	<0.0001
	Model III	1.44 (1.16, 1.78)	0.0024

## Discussion

This investigation conducts a thorough examination of a substantial cohort of U.S. adults diagnosed with diabetes or prediabetes, revealing a pronounced association between the TyG index and the prevalence of HTN. Elevated TyG index readings correlate with an increased probability of HTN development. Notably, participants lacking stroke history demonstrate a more robust linkage, underscoring the influence of prior stroke on this relationship. Furthermore, this study accentuates the substantial relation between various TyG-related metrics and HTN risk, especially considering different obesity indices such as BMI, WC, and WHtR.

The current dearth of HTN predictive tools makes people less aware of their own risk profiles, which in turn makes it harder to effectively manage the condition [31]. IR is is a critical precursor to HTN in individuals with T2D. Although the hyperinsulinemic-euglycemic clamp is considered the definitive method for assessing IR, its complexity and invasive nature limit its practicality for broad clinical application [32]. Another option is to use the Homeostasis Model Assessment (HOMA) index, which is widely used in study and diagnosis of metabolic syndrome and diabetes, to assess IR and β-cell function. This index derives its values from fasting glucose and insulin levels. Elevated HOMA-IR values signify pronounced IR, with figures surpassing 2.9 indicating significant IR. Recently, HOMA-IR has gained acceptance as a feasible proxy for IR assessment [33]. Nonetheless, obtaining IR measurements through insulin-based tests like HOMA-IR remains challenging in resource-limited settings. On the contrary, the TyG index, calculated from readily available clinical data, is adaptable for both advanced and developing regions. It has been recognized as a sensitive and specific biomarker for diagnosing IR, offering greater predictive accuracy than traditional criteria [34][35][36].

The TyG index has been extensively studied in clinical settings for its practical utility. Numerous studies have explored its association with HTN [37][38][39], but results have varied among different populations [40][41][42][43]. Several studies have linked IR to the onset of HTN in individuals with diabetes or prediabetes [44]. A recent prospective and observational clinical research conducted in China indicated that patients with HTN and CHD were more likely to experience adverse events when the TyG index was high [45]. Zheng et al. [37] tracked 4, 686 adults (ranging in age from 20 to 80) for a period of nine years to determine the correlation between the TyG index and the risk of HTN. They noted that it might be used to predict the risk of HTN in the Chinese population and found that a higher TyG index was associated with a higher possibility of developing HTN in the future [46]. As per the study conducted by Chen et al. [47], the association between TyG and the likelihood of developing HTN varied among different groups of waist circumferences. Higher TyG indices were positively related with HTN risk in pre-centrally obese individuals. While there was no linkage between normal waist circumference and HTN risk, centrally obese people had a negative association between a higher TyG index and HTN risk [46]. In their study, Dong et al. [48] compared the predictive power of the TyG index with that of cholesterol and glucose parameters and looked into the correlation between the two using data from the China Health Checkup Epidemiology CHEC Study that ran from 2014 to 202l. Furthermore, it was observed that the TG/HDL-C ratio, TC, and LDL-C each demonstrated a positive correlation with HTN risk. However, their predictive capacities were not as strong as those exhibited by the TyG index [47]. Our findings support existing literature, demonstrating that the TyG index is consistently higher in hypertensive subjects within the diabetes and prediabetes cohort, compared to their non-hypertensive counterparts. This supports the utility of the TyG index as an effective indicator associated with HTN prevalence in this demographic.

Compared to the general population, the percentage of patients with DM who have high BP or use antihypertensive medication is significantly greater, at around 69.0% [48]. Multiple factors contribute to HTN development in these individuals, including increased activity of angiotensin II (AngII) and aldosterone in tissues [49][50], heightened sympathetic nervous system activity [51], and elevated oxidative stress levels. HTN emerges when insulin's action on renal sodium reabsorption enhances, leading to augmented salt and water retention, which subsequently elevates blood volume and pressure. Additionally, IR hinders renal sodium excretion, further intensifying sodium retention and raising BP This condition also affects the renin-angiotensin-aldosterone system (RAAS), which then amplifies BP by boosting the system's overall activity [52]. In addition, IR has the ability to boost endothelin production, which causes the blood vessels to constrict, and decrease prostacyclin (PGI2) and prostaglandin E2 (PGE2) production, which causes the smooth muscle of the blood vessels to proliferate and raise BP [53]. Chronic low-grade inflammation is strongly associated with IR. Damage to vascular endothelium, promotion of atherosclerosis, and elevation of BP can result from high levels of inflammatory chemicals like tumor necrosis factor-alpha, transforming growth factor-β, and reactive oxygen species. These factors can also activate the central nervous system, intensifying sympathetic nervous system activity [54]. Metabolic syndrome, which encompasses obesity, dyslipidemia, hyperuricemia, and hyperglycemia, is another hallmark of IR. These elements jointly heighten HTN risk. Obesity, for instance, increases BP by raising blood volume and activating the sympathetic nervous system, while dyslipidemia and hyperglycemia aggravate HTN through vascular dysfunction and inflammatory response enhancement [55]. More and more research is showing that the TyG index is a reliable indicator of both diabetes and impending HTN; this study adds to that body of evidence. Our findings on the association between the TyG index and HTN suggest that early assessment of the TyG index might help in the identification of individuals at high risk for HTN, which could inform targeted prevention strategies. Based on our findings and the results of future research, we recommend using the baseline TyG index to identify those at high risk of developing HTN at an early stage. Because the TyG index accounts for fasting glucose and triglyceride levels, additional studies are required to determine whether or not individualized pharmaceutical treatments might enhance outcomes.

BMI and WC are commonly used as simple, practical, and non-invasive anthropometric measurements to assess overall body fat and serve as indicators of obesity and metabolic risk [56]. However, they may not effectively assess the accumulation of abdominal fat. The WHtR index is considered a more precise indicator of central obesity, or abdominal fat accumulation [57][58]. Research has shown that metabolic syndrome can be better predicted when the TyG Index is used in conjunction with other obesity indices, such as weight and waist size [25][56]. There is a favorable correlation between the risk of HTN and parameters like TyG-WHtR and TyG-WC that combine the TyG Index with obesity indices including BMI, WC, and WHtR. Therefore, future assessments of HTN risks must include thorough studies that carefully analyze the overall and individual effects of the TyG Index. The purpose of this research is to improve the basis for clinical assessment by clarifying the combined impacts of obesity indices and the TyG Index on HTN risk.

Moreover, our finding of a particularly strong association between the TyG index and hypertension risk in Mexican Americans warrants deeper consideration. This observation is likely rooted in the unique metabolic phenotype frequently described in this population. Extensive research has shown that, compared to non-Hispanic whites, Mexican Americans exhibit a higher genetic and acquired predisposition to insulin resistance (IR), type 2 diabetes, and central obesity [59]. Indeed, clinical evidence indicates that the prevalence of visceral obesity among Mexican Americans can reach 69% even in individuals with a normal BMI, compared to just 31% in non-Hispanic white counterparts, a key driver of metabolic dysfunction [60]. This heightened susceptibility is likely multifactorial. For instance, genetic factors play a significant role; the 5-SNP risk haplotype of the SLC16A11 gene, which is significantly associated with an increased risk of diabetes (OR=1.17), is found at a frequency of up to 30% in individuals of Mexican ancestry. This variant may interfere with normal hepatic lipid metabolism by altering the expression of the SLC16A11 gene or the function of its encoded protein, thereby promoting IR and exacerbating the development of type 2 diabetes [61]. Furthermore, socio-environmental factors, including dietary acculturation - a shift towards processed, high-glycemic-index foods - can exacerbate this underlying genetic predisposition, leading to pronounced dyslipidemia and hyperglycemia [62]. Because the TyG index is calculated directly from fasting triglycerides and glucose, it precisely captures the core metabolic disturbances that are particularly prominent in the pathophysiology of cardiometabolic diseases in Mexican Americans. Therefore, it is plausible that in a population where IR and its downstream effects are a central pathological driver, the TyG index emerges as an especially sensitive and powerful predictor of hypertension. Further research should focus on validating these ethnic-specific risk associations and exploring the underlying mechanisms.

Metabolic processes are vital in illness development, according to recent studies. The heart, brain, and liver are adversely affected by the TyG index, which indicates elevated levels of dyslipidemia and glucose [63][64][65]. Irregular cytokine production, increased acute-phase reactants, and activation of inflammatory pathways are hallmarks of chronic inflammation, which in turn drives lipid imbalances and diabetes, as well as conditions like hyperinsulinemia and IR [54]. Our investigation indicated that the TyG index was positively associated with the risk of HTN in individuals with diabetes or prediabetes who had never experienced a heart attack, stroke, CHD, or liver illness before. Because metabolic instability and IR share risk factors like obesity and inactivity as well as systemic impacts like inflammation and oxidative stress, this correlation may be the result of their intertwined pathophysiological and physiological dynamics [66]. In contrast, those with significant medical backgrounds and high TyG index values might present more severe symptoms rather than a heightened risk of HTN. For patients devoid of significant prior cardiovascular or hepatic conditions but diagnosed with diabetes or prediabetes, the TyG index serves as a valuable tool for early HTN risk detection and intervention. Concurrently, evaluating insulin sensitivity in patients with substantial medical histories is advisable to mitigate the risk of abrupt symptom worsening and to enhance overall health outcomes.

While our research established a positive correlation between the TyG index and HTN risk, several limitations warrant consideration. Primarily, the study's observational nature precluded establishing causality. Thus, additional prospective trials are essential to validate our results. Moreover, although we adjusted for numerous confounding variables, we did not include potential influencers like the specific duration of diabetes, dietary habits, or physical activity levels, due to limitations in data availability and reliability within the dataset. These factors represent potential sources of residual confounding. Furthermore, the applicability of our findings might be restricted to the United States since the study solely involved American adults.

## Conclusions

In this cross-sectional study of patients with type 2 diabetes and prediabetes, the triglyceride-glucose (TyG) index—a simple, reproducible marker calculated from fasting triglycerides and glucose—was independently associated with the prevalence of hypertension. These findings suggest TyG may help improve risk stratification for insulin resistance-related cardiovascular risk, although its prognostic utility should be validated in future longitudinal studies.

## Dodatak

### List of abbreviations

hypertension (HTN); insulin resistance (IR); Triglyceride-Glucose (TyG); National Health and Nutrition Examination Survey (N HAN ES); coronary heart disease (CHD); cardiovascular diseases (CVD); triglycerides (TG); total cholesterol (TC); low-density lipoprotein cholesterol (LDL-C); high-density lipoprotein cholesterol (HDL-C); blood pressure (BP); Diabetes Mellitus (DM); Type 1 Diabetes (T1D); Type 2 Diabetes (T2D); National Center for Health Statistics (NCHS); family income to poverty ratio (PIR); hemoglobin A1c (HbA1c); fasting blood glucose (FBG); refrigerated serum creatinine (SCR); blood urea nitrogen (BUN); standard deviations (SD); myocardial infarction (MI); prostacyclin (PGI2); prostaglandin E2 (PGE2); renin-angiotensin-aldosterone system (RAAS).

### Author contributions

The study design was conceived by Tianyu Dai, Jian Wang, Fangze Wang, Hanqing Deng, Muyuan Li, and Jihu Zhang. TD, FW and JW organized the data, conducted the analyses, and wrote and edited the manuscript.HD, ML, and JZ contributed to the interpretation of the results, revision, and finalization of the manuscript. All authors have reviewed and approved the final version of the manuscript.

### Funding

The authors did not receive support from any organization for the submitted work.

### Ethics statement

The NHANES project has been approved by the NCHS Ethics Review Board (ERB), and all participants have consented to data collection.

### Availability of data and materials

Publicly available datasets were analyzed in this study. This data can be found here: https://www.cdc.gov/nchs/nhanes/index.htm.

### Conflict of interest statement

All the authors declare that they have no conflict of interest in this work.
